# Periostin overexpression in scleroderma cardiac tissue and its utility as a marker for disease complications

**DOI:** 10.1186/s13075-022-02943-2

**Published:** 2022-11-11

**Authors:** Fatima El-Adili, Justin K. Lui, Mortada Najem, Giuseppina Farina, Maria Trojanowska, Flora Sam, Andreea M. Bujor

**Affiliations:** 1 Division of Rheumatology, Boston University School of Medicine, Boston, MA USA; 2 Whitaker Cardiovascular Institute, Boston University School of Medicine, Boston, MA USA; 3 Arthritis and Autoimmune Diseases Center, Boston University, 72 E Concord St, Evans 501, Boston, MA 02118 USA; 4 The Pulmonary Center, Boston University School of Medicine, Boston, MA USA

**Keywords:** Scleroderma, Periostin, Biomarkers, Fibrosis

## Abstract

**Objective:**

To evaluate the levels of periostin in patients with systemic sclerosis (SSc) and their association with features of systemic sclerosis.

**Methods:**

The levels of periostin were assessed in the serum of 106 SSc patients and 22 healthy controls and by immunofluorescence staining in cardiac tissue from 4 SSc patients and 4 controls. Serum periostin was measured via enzyme-linked immunosorbent assay. The results were analyzed using Mann-Whitney test or Kruskal-Wallis test followed by Dunn’s multiple comparisons tests and Spearman’s test for correlations. Cardiac tissue from SSc patients and controls was stained for periostin and co-stained for periostin and collagen type I using immunofluorescence.

**Results:**

Periostin levels were higher in patients with SSc compared to controls and directly correlated to modified Rodnan skin score and echocardiography parameters of left ventricular measurements. Immunofluorescence staining in SSc cardiac tissue showed patchy periostin expression in all SSc patients, but not in controls. Furthermore, there was extensive periostin expression even in areas without collagen deposition, while all established fibrotic areas showed colocalization of collagen and periostin. There was no association between periostin levels and interstitial lung disease, pulmonary hypertension or other vascular complications.

**Conclusion:**

Periostin is elevated in SSc cardiac tissue in vivo and circulating levels of periostin are increased in SSc, correlating with the extent of disease duration, degree of skin fibrosis, and left ventricular structural assessments. Periostin may be a potential biomarker that can provide further pathogenic insight into cardiac fibrosis in SSc.

## Introduction

Scleroderma/systemic sclerosis (SSc) is an autoimmune connective tissue disease characterized by vascular abnormalities and progressive, widespread tissue fibrosis that is associated with poor prognosis and constitutes one of the highest mortalities among autoimmune diseases [1]. Vascular and fibrotic manifestations are central to SSc and can affect almost any organ, but their extent and functional consequences are diverse between patients, accounting for the large heterogeneity in phenotypic expression [2]. With an incomplete understanding of disease pathogenesis, there is currently no uniformly effective treatment for SSc [3]. Immunosuppressive, antifibrotic, and vasodilating agents are the mainstay of therapy, depending on the disease complications, which can range from mild skin involvement to severe, life-threatening complications including interstitial lung disease (ILD), pulmonary hypertension (PH), and cardiac dysfunction from SSc-related cardiomyopathy [4, 5]. One of the challenges that physicians treating SSc patients face is the lack of validated measures to identify patients at risk for progression to severe disease. A biomarker reflecting SSc disease severity and complications would help individualize therapy, potentially preventing progression of organ damage and sparing at-risk patients from serious medication adverse effects [6–9].

Periostin is a secreted 90 kDa matricellular protein expressed by fibroblasts and epithelial cells with important roles in fibrosis, cell adhesion, survival, angiogenesis, and matrix remodeling [10, 11]. It regulates cell-matrix organization and collagen fibrillogenesis, binding to integrins and promoting activation of Akt and FAK-mediated signaling [12, 13]. Expressed in many tissues during fetal development, periostin production is downregulated in adult tissues under physiological conditions, and highly upregulated during injury [10, 14]. Because periostin is easily secreted and can be detected noninvasively in biological fluids, its utility as a biomarker has been investigated in various diseases, including SSc, idiopathic pulmonary fibrosis (IPF), asthma, and cancer. Circulating levels of periostin correlated with disease severity in IPF [15–18] and with the extent of skin thickening in SSc [19]. Immunohistochemistry analyses of periostin expression in SSc skin revealed robust upregulation throughout the dermis in SSc compared to controls, where periostin expression was low and limited to the dermal-epidermal junction [13, 19–21]. Additionally, there was periostin co-localization with alpha smooth muscle actin (ASMA) and platelet and endothelial cell adhesion molecule 1 (PECAM-1), suggesting that myofibroblasts and endothelial cells are the source of increased periostin in SSc skin [19].

Extensive evidence supports the pivotal role of type 2 inflammation in SSc pathogenesis, with increased Th2-polarized cell tissue infiltration and higher levels of circulating IL-4 and IL-13 [22]. Of relevance, periostin is an IL-4/IL-13-inducible gene and a potential surrogate marker of T-helper type-2 (Th2) inflammation [23]. A recent study found that dermal periostin overexpression correlated with macrophage and T lymphocyte infiltration in SSc skin, suggesting a role for periostin in inflammation-induced fibrosis in SSc [20]. Periostin was thus recently used as a biomarker in a proof-of-concept study in patients with SSc to assess the response to Romilkimab therapy (a monoclonal bispecific antibody against IL-4/IL-13), showing a greater trend of reduction compared to placebo [24].

Two studies investigated the potential role of circulating periostin as a biomarker of disease severity in SSc, with contradicting results. While both found elevated expression of periostin in the serum and in the skin of SSc patients compared to controls, one also found a positive correlation of serum periostin with the modified Rodnan skin score (mRSS), the gold standard for clinical assessment of skin fibrosis, while the other failed to validate this additional finding. Surprisingly, despite evidence that periostin might be a useful marker for IPF, these two studies in SSc patients found no association with SSc-ILD [19, 20].

Critical for the development and maturation of cardiac tissues, periostin is highly induced upon injury, and is a specific cardiac myofibroblast marker, essential in the pathogenesis of cardiac fibrosis [25, 26]. Periostin-null mice are resistant to cardiac fibrosis, while inducible periostin-overexpressing mice develop spontaneous hypertrophy with aging [27, 28]. To date, no association between periostin and cardiac complications in SSc has been described.

Our current knowledge of the role that periostin plays in many fibrotic processes, together with encouraging, albeit preliminary data from SSc patients, suggests that periostin may be a promising biomarker of disease complications in SSc. This study was undertaken to further elucidate the role of circulating periostin as a biomarker for SSc organ complications. Using SSc patients from our cohort at Boston University Medical Center, we showed that circulating periostin associated with disease duration and correlated to the extent of skin fibrosis as measured by the mRSS. We also confirmed previously published results showing a lack of association between periostin levels and SSc-ILD and SSc-PH. For the first time, we report that periostin is expressed at high levels in SSc cardiac tissue in vivo, even in areas without significant collagen deposition. Furthermore, levels of circulating periostin correlated to echocardiographic measurements of left ventricular size. Collectively, these results implicate periostin in the process of skin and cardiac fibrosis in SSc, suggesting that it may be a promising biomarker to assess for these organ-specific SSc complications and guide future therapies. The scope of this study was to investigate whether periostin may be applied to subtype SSc patients based on disease manifestations.

## Materials and methods

### Study population and clinical characteristics

Serum samples and associated clinical and laboratory data from 106 SSc patients, enrolled in an existing database at Boston University School of Medicine (SCaR clinical database), were used to measure the levels of circulating periostin. Clinical and laboratory evaluations were performed in the Scleroderma Clinic at Boston Medical Center, from 2002 to 2020. All subjects consented to participate in a research study under a protocol approved by the Boston University Medical Center Institutional Review Board. All SSc patients fulfilled the new ACR/EULAR 2013 classification criteria and were classified as limited (lcSSc) or diffuse SSc (dcSSc) according to LeRoy classification [29, 30]. Demographics and data on clinical features were collected which included (1) disease duration (defined by onset of first non-Raynaud’s disease manifestation); (2) SSc complications (digital ulcers, pitting scars, telangiectasias, joint contractures, ILD, and PH); and (3) severity of skin disease by modified Rodnan skin score (mRSS). ILD was determined by high-resolution computed tomography. PH was defined on right heart catheterization by a mean pulmonary arterial pressure (mPAP) > 20 mm Hg with a pulmonary arterial wedge pressure (PAWP) ≤ 15 mm Hg and pulmonary vascular resistance (PVR) ≥ 3 Wood units, as per the new Sixth World Symposium on Pulmonary Hypertension criteria [31]. Additionally, we compiled data on clinical assessments consisting of (1) pulmonary function testing (forced vital capacity [FVC], forced expiratory volume in 1 second [FEV_1_], and diffusing capacity of carbon monoxide [D_LCO_]; (2) echocardiography measurements (left ventricular ejection fraction [LVEF], left atrial diameter [LA], interventricular septal diameter [IVS diameter], posterior wall diameter [PW diameter], left ventricular end-diastolic diameter [LVEDD], left ventricular end-systolic diameter [LVESD], fractional shortening [FS], left ventricular mass [LV mass], and left ventricular mass index [LV mass index]); and (3) laboratory studies (brain natriuretic peptide [BNP]). The clinical assessments closest to the date of sample collection was used for analysis.

### Biochemistry and immunochemistry assays

#### ELISA measurements

Serum levels of periostin were measured using a human periostin ELISA kit (EHPOSTN, Thermo Fisher Scientific, Waltham, MA) at 1:100 dilution in duplicates according to manufacturer’s protocol. Absorbance was measured on a microplate reader (Multiskan EX, Thermo Scientific, Vantaa, Finland).

#### Immunohistochemistry and Immunofluorescence of human cardiac tissue

Paraffin embedded human cardiac tissue was obtained from National Disease Research Interchange from SSc patients and controls. Two of the SSc cardiac tissue samples were obtained in collaboration with Dr. Farina, from deceased patients at the Department of Immunology, La Sapienza University, Rome, Italy. Formalin-fixed, paraffin embedded blocks were sectioned at 5-μm thickness and stained for periostin (red) with counterstaining for nuclei with 4′,6-diamidino-2-phenylindole (DAPI). Briefly, slides were deparaffinized and fixed with acetone/methanol and then blocked with 1% bovine serum albumin (BSA) for 1h. Primary antibody (rabbit polyclonal anti-periostin, Abcam 14041, Cambridge, MA) was added overnight at 4 °C and then washed with phosphate buffered saline and incubated with Alexa Fluor 594 donkey anti-rabbit antibody (ThermoFisher Scientific, A21207, Waltham, MA) for 1 h at room temperature. After three washes, DAPI was used to counterstain the nuclei using Vectashield® Antifade Mounting Media (Vectorlabs, Burlingame, CA). For double staining for collagen type I and periostin, tissues were sent to Servicebio (Woburn, MA) and stained using the following antibodies: Anti-type I collagen (rabbit polyclonal GB11022-1, Servicebio, Woburn, MA), and periostin (Abcam 14041, Cambridge, MA). Staining was examined using a FluoView FV10i confocal microscope system (Olympus, Center Valley, PA, USA).

For immunohistochemistry, the blocks were sectioned at a thickness of 5 μm, and the tissue sections were deparaffinized. After antigen retrieval with 10 mM sodium citrate buffer, the slides were blocked in 3% BSA for 1 h, and incubated overnight with antibodies against alpha smooth muscle actin (#19245, Cell Signaling, Danvers, MA, at 1:100 dilution), followed by the incubation with secondary antibody ImmPRESS AP, Horse Anti-Rabbit MP-5401, Vectorlab, Burlingame, CA) for 20 min. The immunoreactivity was visualized with using the Vector blue substrate kit AP (SK-5300, Vectorlab, Burlingame, CA). The slides were rinsed in wash buffer and subsequently incubated with collagen type I (Southern Biotech 1310-01, Birmingham, AL, 1:50 dilution) for 1 h at RT, followed by the incubation with secondary antibody (ImmPRESS HRP, Horse Anti-Goat MP-7405, Vectorlab, Burlingame, CA) for 20 min. The immunoreactivity was visualized using ImmPACT DAB HRP (SK-4103, Vectorlab, Burlingame, CA). Slides were mounted with aqueous mounting medium and visualized under microscope.

### Statistical analysis

Continuous variables were described by the mean (± standard deviation [SD]), and categorical variables were described by their frequencies and percentages for each group. As data did not follow a normal distribution, non-parametric tests were used for analyses. Three samples showed very high periostin levels and were considered outliers and removed from analyses. A Kruskal-Wallis test followed by Dunn’s multiple comparisons tests was used to compare differences between three or more groups. Mann-Whitney test was used when only two groups were compared. Correlations between periostin and other parameters were evaluated by Spearman’s correlation coefficients. All statistical analyses were performed using GraphPad Prism 9 software (La Jolla, CA, USA). A *p* value of < 0.05 was considered significant.

## Results

### Clinical characteristics

A total of 106 patients with SSc were included in the analyses, 83% of which were women, with a mean age of 55.7 years, and a mean disease duration of 12.21 ± 2 years (as defined by onset of first non-Raynaud’s disease manifestation). Seventy-seven (72.6%) patients had lcSSc, and 29 (27.4%) patients had dcSSc (Table 1). The frequency of ILD as determined by high resolution computed tomography was 45.3%. Precapillary PH was present in 31.13% of patients. The mean mRSS was 9.86.

**Table 1:** Baseline characteristics for SSc patients

Characteristics	SSc (***n*** = 106)
Age, mean ± SD	55.7 ± 12.22
Sex: female	88
Diffuse SSc	29
Limited SSc	77
Disease duration^†^ (years, mean ± SD)	12.21 ± 22.94
ILD	48 (45.28%)
Pulmonary arterial hypertension*	33 (31.13%)
MRSS, mean ± SD	9.86 ± 11.03
Digital ulcers, past or current	29/67 (43.28%)
Pitting scars	25/63 (39.68%)
Teleangiectasias	27/53 (50.94%)
joint contractures	14/57 (24.56%)
Current/former smoker	41/92 (44.57%)
Respiratory function tests, mean ± SD	
FVC (% predicted)	74.89 ± 19.54
FEV1 (% predicted)	75.47 ± 19.35
DLCO (% predicted)	49.74 ± 19.95
LVEF (%)	57.19 ± 9.25
LA (mm)	34.47 ± 5.22
IVS (mm)	9.063 ± 1.59
PW (mm)	8.91 ± 1.58
LVEDD (mm)	44.50 ± 5.60
LVESD (mm)	29.47 ± 5.74
FS	34.08 ± 7.66
LV mass (g, ASE)	131.30 ± 40.27
LV mass index (g/m^2^)	72.16 ± 20.05

^†^Defined as the onset of the first non-Raynaud’s manifestation; information only available for 68/106 patients

^*^Defined as mPAP >20 mmHg, PVR>240 dynes/sec/cm−5 and PCWP ≤ 15 mmHg

### Association with disease subtype and duration

**Fig. 1:**
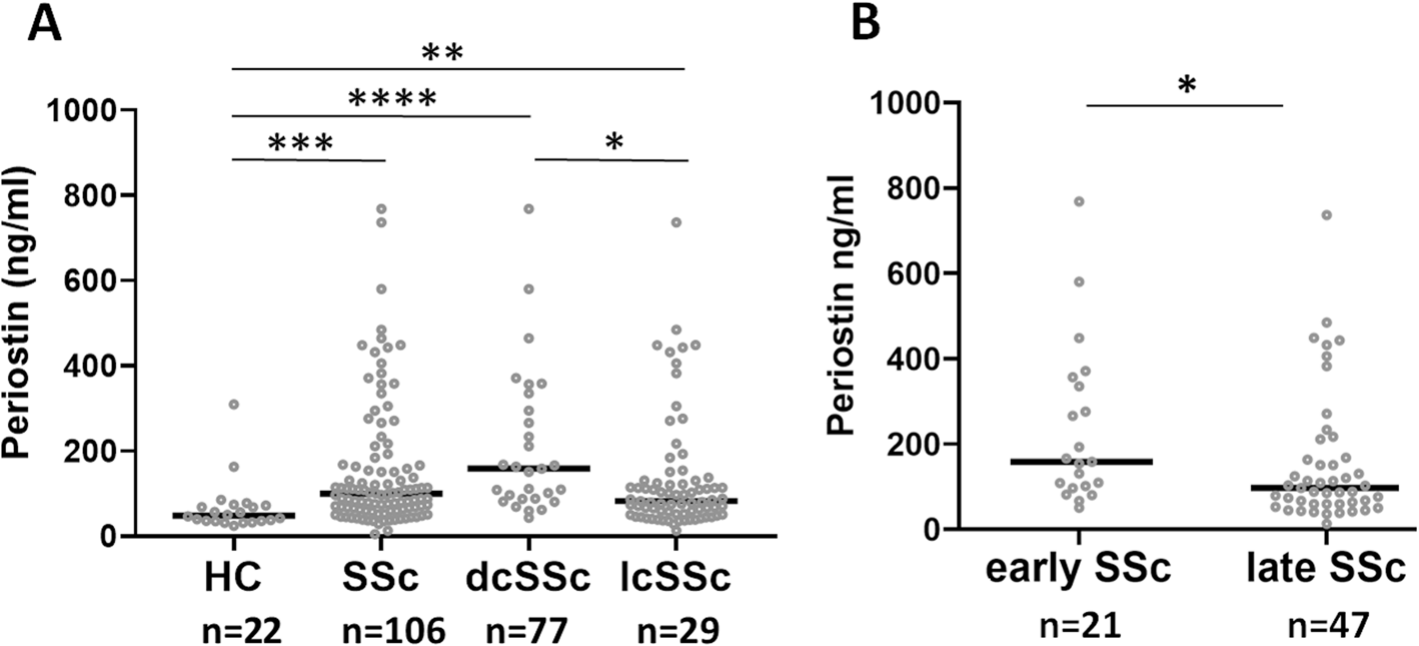
Differences in serum levels of periostin based on disease type and duration. Scatterplot showing distribution of serum periostin levels in controls (HC), dcSSc and lcSSc patients in **A** and in early (under 3 years) or late (over 3 years) SSc. Bars represent mean and standard deviation, analyzed by Kruskal-Wallis test with Dunn’s multiple comparisons test in **A** and unpaired Mann-Whitney test in **B** (GraphPad Prism 9); *p* < 0.05 *, *p* < 0.01**, *p* < 0.001***, *p* < 0.0001****

Levels of periostin where measured in patients with SSc and periostin expression was compared between SSc subgroups and with a healthy control group. There was a statistically significant difference in the levels of periostin between groups as determined by Kruskal-Wallis test (*p* < 0.0001). A Dunn’s multiple comparisons analysis revealed that periostin levels were statistically significantly higher in the dcSSc group (211.9 ± 172.3 ng/ml) compared to both the control group (66.97 ± 61.72 ng/ml, *p* < 0.0001) and the lcSSc group (130.9 ± 133.7 ng/ml, *p* = 0.01). There were also significantly higher levels between lcSSc and the control group (*p* = 0.0071), as seen in Fig. 1A.

Next, we assessed the effect of disease duration on serum periostin in SSc patients using Mann-Whitney test in two patient groups: early SSc (disease duration under 3 years) and late SSc (disease duration over 3 years). As seen in Fig. 1B, early SSc patients had higher levels of periostin than patients with late SSc (233.3 ± 187.5 vs 154.7 ± 152.6, *p* = 0.02). Periostin levels were also correlated to disease duration by Spearman’s correlation coefficient (Table 2).

**Table 2:** Correlation analysis between serum periostin and continuous clinical variables in SSc patients

Parameter	Number of pairs	***R*** coefficient	***p*** value
Age	106	− 0.07	0.46
Disease duration	71	− 0.28	**0.018**
mRSS	81	0.49	**< 0.0001**
FVC	92	− 0.14	0.18
FEV1	93	− 0.15	0.16
DLCO	89	− 0.11	0.29
BNP (pg/l)	60	0.17	0.20

Spearman’s correlation analysis showing correlation between periostin (ng/ml) and disease duration, and mRSS in SSc patients. *mRSS* Modified Rodnan skin score, *FVC* Forced vital capacity, *FEV1* Forced expiratory volume, *DLCO* Diffusing capacity for carbon monoxide, *BNP* Brain natriuretic peptide

### Association with skin fibrosis and ILD

**Fig. 2:**
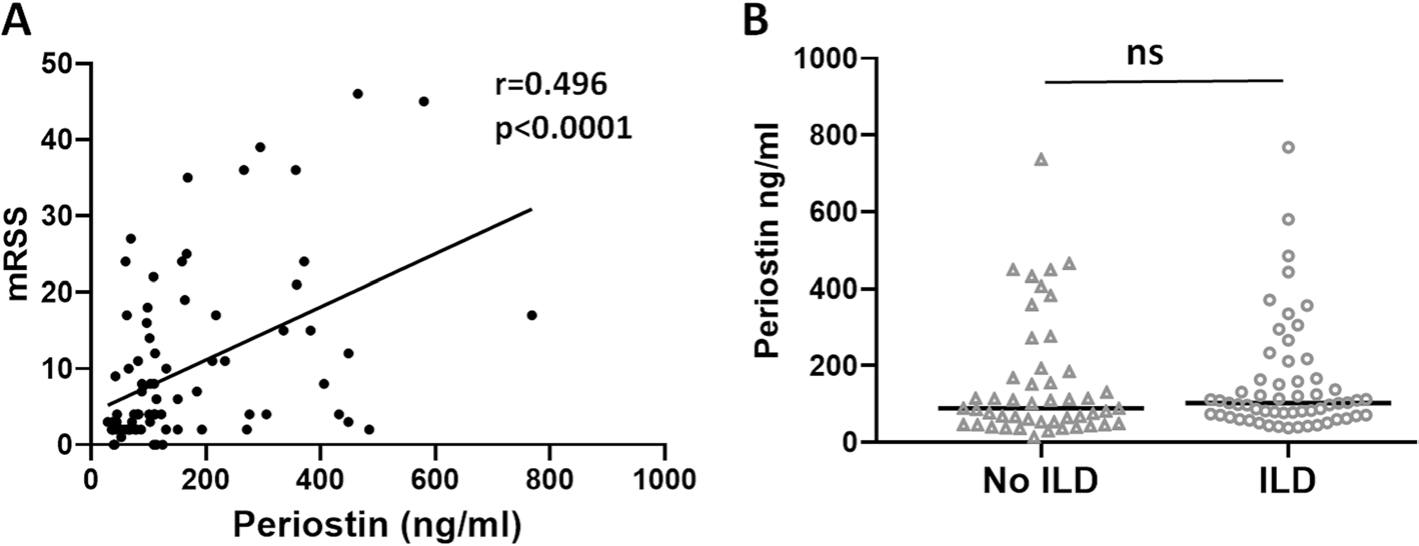
Periostin directly correlates with MRSS but is not increased in SSc-ILD. Spearman’s correlation analysis showing direct correlation between MRSS (modified Rodnan skin score) and circulating periostin; *r*-Spearman correlation coefficient; number of XY pairs: 81; *p* < 0.0001****. Scatterplot showing distribution of serum periostin levels in SSc patients without and with ILD. Bars represent mean and standard deviation, analyzed by unpaired Mann-Whitney test; ns, not significant

We next sought to evaluate the potential correlation between periostin levels and the extent of skin fibrosis as measured by mRSS. There was a direct correlation between mRSS and periostin levels as seen by Spearman’s correlation analysis (Fig. 2A).

Given the important roles that periostin may play in lung fibrosis, we sought to further explore its expression in relationship with SSc-ILD. However, there were no differences in periostin levels between SSc patients with and without ILD (153.1 ± 144.1 vs 153.1 ± 156.0, *p* = 0.40, Fig. 2B). Furthermore, by pulmonary function testing, periostin did not show a strong correlation to FVC, FEV_1_, and D_LCO_ (Table 2). Of note, only 5 patients without ILD had the dcSSc subtype (8.6%), compared to 24 patients with ILD (50%). The mean mRSS in patients with ILD was approximately double that of no ILD patients (12.84 ± 11.59 vs 6.63 ± 9.58, *p* = 0.01). These results suggest that periostin levels do not correlate with the fibrotic lung complications in SSc.

### Association with vascular complications

Vascular injury is central and represents an initial event in the pathogenesis of SSc [32]. We next investigated the possible relationship between circulating periostin and SSc vascular complications. There were no differences in periostin levels between patients with and without (1) telangiectasias, (2) digital ulcers, and (3) pitting scars (Table 3). Next, data from right heart catheterization was used to identify patients with precapillary pulmonary hypertension, as defined in the “Materials and methods” section. A total of 33 patients (31.13%) met the criteria for SSc-PH. Periostin levels in this patient group were compared to the levels in the remaining patients. There was no difference in periostin levels between patients with and without SSc-PH (156.6 ± 143.4 versus 145.4 ± 162.3, *p* = 0.47). Based on these results, there is no evidence to support a role for periostin as a biomarker for vascular complications of SSc.

**Table 3:** Comparison of serum periostin levels according to categorical clinical variables in SSc patients

Variable	***N***	periostin (ng/ml))	***p*** value
Sex			
F	88	133.0 ± 128.0	**0.005**
M	18	251.5 ± 201.6	
Disease subtype			
lcSSc	77	130.9 ± 133.7	**0.001**
dcSSc	29	211.9 ± 172.3	
ILD			
Y	58	153.1 ± 144.1	0.40
N	48	153.1± 156.0	
PAH			
Y	33	145.4 ± 162.3	0.47
N	73	156.6 ± 143.4	
Digital ulcers, past or current			
Y	14	230.5 ± 175.9	0.22
N	45	154.0 ± 149.8	
Pitting scars, past or current			
Y	25	174.6 ± 167.1	0.75
N	38	154.7 ± 145.1	
Teleangiectasias			
Y	27	161.1 ± 171.1	0.35
N	26	190.9 ± 154.3	
Current/former smoker			
Y	41	160.2 ± 162.2	0.96
N	51	142.4 ± 137.4	
Joint contractures			
Y	14	220.7 ± 165.7	0.06
N	43	140.8 ± 125.2	

*F* Female, *M* Male, *N* Number of patients with available information, *lcSSc* Limited scleroderma, *dcSSc* Diffuse scleroderma, *ILD* Interstitial lung disease, *PAH* Pulmonary arterial hypertension, *Y* Yes, *N* No. Data are presented as mean ± standard deviation. Mann-Whitney test was used for analyses

### Association of circulating periostin with other patient- or disease-related features

Serum periostin levels were compared for various categorical and continuous variables, using Mann-Whitney test or Spearman’s correlation analyses respectively. Male patients had higher periostin levels than female (251.5 ± 201.6 vs. 133.0 ± 128.0, *p* = 0.005, Table 3). Patient with joint contractures also had higher periostin levels, but the results did not reach statistical significance (220.7 ± 165.7 vs. 140.8 ± 125.2, *p* = 0.06, Table 3).

### Correlations between periostin and parameters related to cardiac involvement

**Fig. 3:**
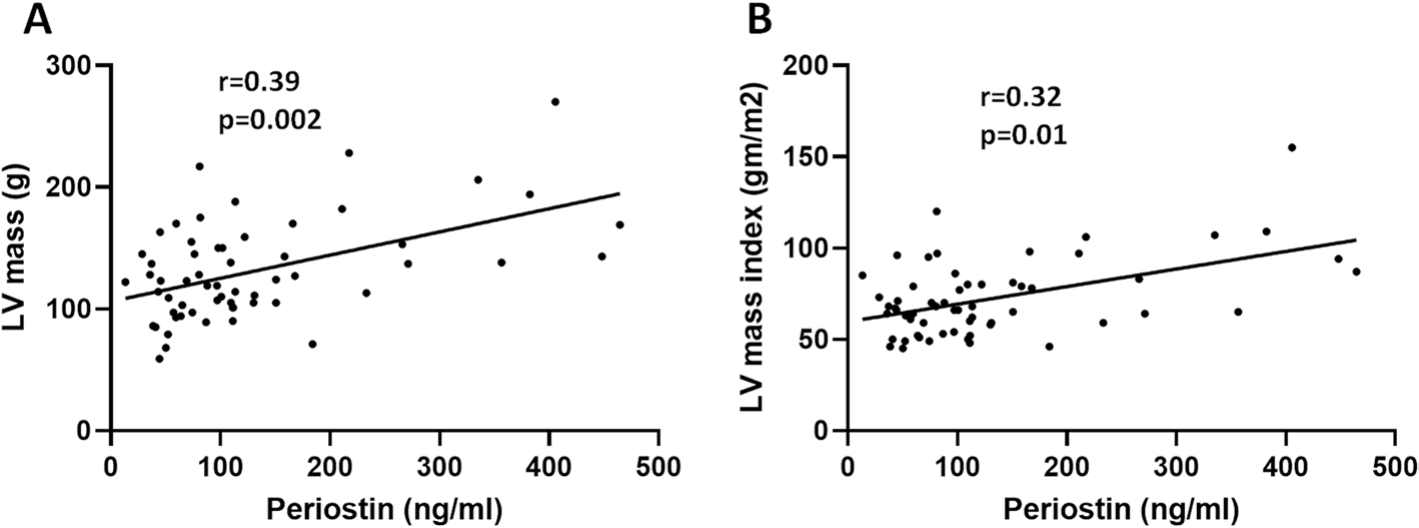
Direct correlation between periostin and left ventricular mass and left ventricular mass index. Spearman’s correlation analysis showing direct correlation between LV mass, LV mass index (LV mass/BSA) and circulating periostin; *r*-Spearman correlation coefficient; number of XY pairs: 59 for both; *p*, *p* value. LV, left ventricle, BSA, body surface area

Cardiac fibrosis leading to conduction abnormalities and diastolic dysfunction is being increasingly recognized as an important risk factor for mortality in SSc. Subclinical cardiac fibrosis is difficult to diagnose without a cardiac biopsy, which is rarely performed in SSc. We next performed correlation analyses between circulating periostin levels and echocardiographic measurements of left ventricular (LV) dimensions, for all patients that had at least one echocardiogram performed at Boston Medical Center (*n* = 59). As seen in Fig. 3, there was significant correlation between periostin and LV mass (*r* = 0.39, *p* = 0.002) and LV mass index (*r* = 0.32, *p* = 0.01).

Furthermore, periostin directly correlated with other echocardiographic parameters, including interventricular septum (IVS), LV posterior wall thickness (PW), and LV end diastolic and end systolic diameters (LVEDD and LVESD) as well as left atrial diameter (Table 4).

**Table 4:** Correlations between serum periostin and echocardiographic parameters in SSc patients

Echocardiographic parameters (***n*** = 59)	***R*** coefficient	***p*** value
LVEF (%)	− 0.13	0.32
LA (mm)	0.32	**0.01**
IVS (mm)	0.28	**0.03**
PW (mm)	0.32	**0.01**
LVEDD (mm)	0.29	**0.03**
LVESD (mm)	0.29	**0.03**
FS	− 0.11	0.43
LV mass (g, ASE)	0.39	**0.002**
LV mass index (g/m^2^)	0.32	**0.01**

Spearman’s correlation analysis showing direct correlation between circulating periostin and LA, PW, LVEDD, LVESD, LV mass and LV mass index; *LVEF* Left ventricular ejection fraction, *LA* Left atrium, *IVS* Interventricular septum, *PW* Left ventricular posterior wall thickness, *LVEDD* Left ventricular end diastolic dimension, *LVESD* Left ventricular end systolic dimension, *FS* Fractional shortening, *LV* Left ventricle

Brain natriuretic peptide (BNP) is a hormone primarily released by the ventricles in response to the high filing pressures and wall stress in patients with heart failure. Circulating BNP levels are routinely used in clinical practice for the diagnosis and monitoring of the patients with clinical heart failure, but their levels do not change in asymptomatic systolic or diastolic dysfunction. In this study, we found a weak positive correlation between periostin and BNP levels, which did not reach statistical significance (*r* = 0.22, *p* = 0.08). Similarly, the degree of shortness of breath as assessed by NYHA functional classification did not correlate with the levels of periostin (*p* = 0.138)*.*

### Altered expression of periostin in SSc cardiac tissue

**Fig. 4:**
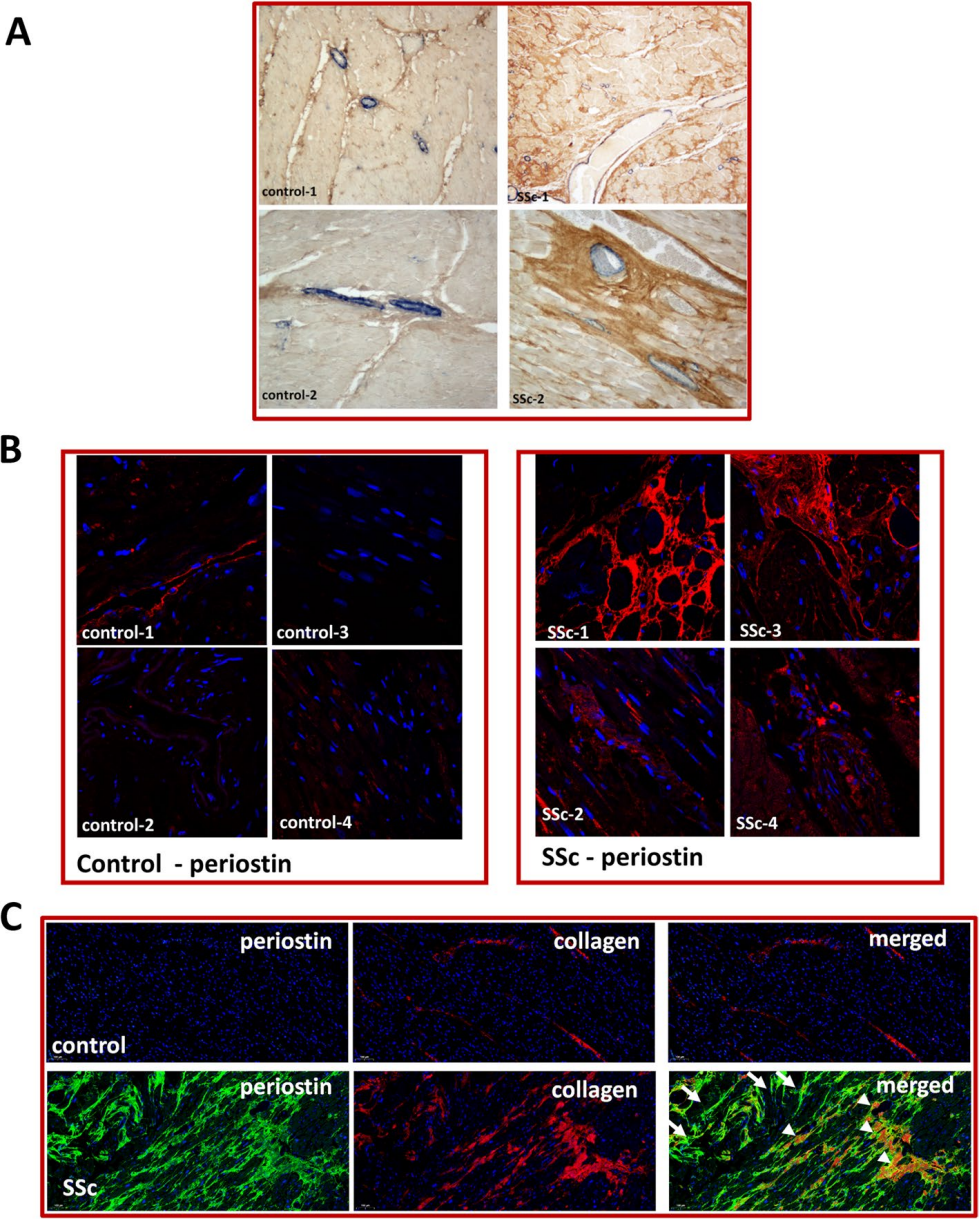
Periostin overexpression in SSc cardiac tissue. **A** Immunohistochemistry in paraffin embedded heart tissue from SSc patients and controls; note ASMA expression in blue restricted to capillaries and small blood vessels in both SSc and control hearts, with enhanced interstitial collagen deposition in SSc (brown); *n* = 2 each, representative sections shown. **B** Immunofluorescence staining for periostin (red) and DAPI (nucleus, blue) in control and SSc hearts, *n* = 4 each, representative sections shown. **C** Immunofluorescence staining for periostin (green) and collagen type I (red) in control and SSc hearts. Nuclei were stained with DAPI (blue). Arrowheads indicate periostin staining that colocalized with collagen type I, and arrows show isolated periostin staining. *n* = 2 each, representative sections shown. Control-1: F, 68 years old (y.o.); Control-2: F, 70 y.o.; Control-3: M, 40 y.o.; control-4: F, 46 y.o.; SSc-1: M, 56 y.o.; SSc-2: F, 80 y.o.; SSc-3 and 4: information not available

Circulating biomarkers are attractive due to the availability of samples and ease of quantification, but they may not reflect what happens locally in the tissue. To assess if periostin is elevated in SSc hearts, we investigated the periostin levels in vivo in heart samples form patients with systemic sclerosis and controls via immunofluorescence. As seen in Fig. 4B, all hearts from SSc patients exhibited positive, patchy staining for periostin, while there was no or minimal periostin expression in control hearts. This was in contrast to alpha smooth muscle actin (ASMA), which was mainly expressed in the capillaries and small blood vessels in both SSc and control hearts, despite obvious interstitial fibrosis as seen by staining for collagen type I in SSc (Fig. 4A).

Since periostin was previously linked to fibroblast activation in the heart, we wanted to assess if its localization in SSc hearts parallels that of tissue fibrosis. We used immunofluorescence to stain for periostin and collagen and note that all areas with collagen type I displayed extensive periostin expression. Interestingly, periostin was also upregulated in many areas of the heart that were not yet affected by fibrosis, suggesting that deposition of periostin may be an early event in fibroblast activation in SSc-CMP (Fig. 4C). Altogether, these results suggest that periostin could be a marker of activated fibroblasts in SSc CMP.

## Discussion

SSc is a heterogeneous systemic disease with various organ manifestations and complications. Vascular injury in SSc can cause ischemic tissue damage or vascular remodeling leading to PH or SSc renal crisis. Activation of fibroblasts with excessive deposition of extracellular matrix (ECM), disrupts normal tissue architecture, resulting in progressive skin thickening, flexion contractures, and fibrosis of the lung and cardiac tissues. The spectrum of organ involvement is variable from patient to patient, and the rate of progression in an individual patient is difficult to predict. Due to this large degree of phenotypic variance, there is currently no single biomarker that can predict SSc disease activity or severity. Different, albeit likely overlapping pathophysiological processes, may contribute to the various disease manifestations in SSc. Understanding which patient is at risk for specific organ involvement early in the disease is vital, as timely therapeutic intervention may prevent disease progression and increase survival [33, 34].

Periostin plays important roles in wound healing, ECM deposition, and tissue fibrosis. Animal studies using periostin-null mice suggest an important role for periostin in wound healing and fibrosis [35, 36]. Highly induced by Th2 cytokines (IL4, IL13) and TGFβ, periostin binds to various integrins and induces fibrosis via TGFβ and PI3K/Akt-dependent pathways [13, 37]. Periostin also contributes to collagen fibrillogenesis and cross-linking, via its interactions with other ECM components, and loss of periostin in mice resulted in reduced collagen fiber diameter and strength [38]. In the bleomycin-induced SSc mouse model, periostin was abundantly expressed in the fibrotic skin and lungs, and knockout of periostin markedly attenuated the degree of sclerotic changes [13, 39, 40]. Analyses of lesional skin of SSc patients demonstrated high levels of periostin expression, particularly in the areas of fibroblast proliferation and immune cell infiltration [13, 19, 20]. These findings suggest a pathogenic role for periostin in fibrosis in SSc, which may prompt further investigations into its utility as a circulating biomarker of SSc fibrotic complications.

The role of periostin in SSc is controversial with respect to its pathophysiologic implications in skin and pulmonary fibrosis. Increased circulating periostin levels have been reported in SSc skin fibrosis [19], although this has not been reproduced by a later report [20]. Our study addressed this inconsistency. Using a larger number of SSc patients compared to previous studies, we found that periostin levels strongly correlated to the degree of skin fibrosis as measured by mRSS. In the study by DeLuca et al, the lack of correlation may be due to poor statistical power secondary to a smaller number of patients included [20].

Previous studies suggest that periostin may play a critical role in the pathogenesis of pulmonary fibrosis and may serve as a biomarker of disease activity and progression in IPF [15, 18, 39]. SSc-ILD and IPF are some of the most common fibrotic lung diseases, sharing some overlapping pathogenetic features [41]. Despite some degree of overlap, there are significant differences between IPF and SSc-ILD, with important clinical implications. The disease in IPF is limited to the lungs and characterized by usual interstitial pneumonia (UIP) on high resolution CT. Patients with SSc-ILD have other organ involvement, and non-specific interstitial pneumonia (NSIP) is most commonly seen on imaging. Demographic differences also exists, with IPF mainly affecting older white males [42], while SSc-ILD is seen predominantly in younger, African-American females [43, 44]. Immunosuppressive therapy has been the mainstay of treatment in SSc-ILD, but it is not indicated and may even worsen IPF [45]. While periostin has been linked to IPF, two previous studies in SSc failed to show any association between serum periostin levels and the presence of pulmonary fibrosis/ILD, although the number of patients was rather small in both studies [19, 20]. Although one study did find an inverse correlation with vital capacity in SSc-ILD, the latter did not reach statistical significance [19]. Similarly, we found no significant difference in circulating periostin between SSc patients with ILD and without ILD. This was despite higher mRSS scores and greater percentage of diffuse SSc patients in the ILD group (two of the main disease features that correlated with high periostin levels). These results further support the notion that periostin is not a reliable biomarker for SSc-ILD and underscore the differences between lung fibrosis in IPF and SSc-ILD. Whether this lack of association with SSc-ILD stems from different pathogenetic mechanisms or is related to other disease specific characteristics (demographics, involvement of multiple organs, etc.) remains to be determined.

Vascular damage is seen almost universally in SSc patients and is an important contributor to SSc pathogenesis. Periostin may also be involved in the response of the vessel wall to injury, being dramatically and transiently overexpressed by smooth muscle cells of the neointima and the adventitial myofibroblasts in balloon-injured rat carotid arteries [46]. Periostin expression was increased in pulmonary smooth muscle cells after hypoxic injury [47], and periostin was previously found to be elevated in patients with PH [48]. In the study by DeLuca et al., SSc patients with active nailfold capillaroscopic pattern and history of digital ulcers were found to have higher circulating levels of periostin. However, there were no other associations with disease subtype or clinical features observed [20]. Nevertheless, in our current study, circulating periostin did not correlate with PH or other SSc related vascular complications, including digital ulcers or telangiectasias. Future studies in larger patient populations will be needed to clarify these inconsistencies.

Widespread cardiac fibrosis is the hallmark of SSc cardiomyopathy (SSc-CMP), affecting up to 70% of patients. Determining who will develop cardiac fibrosis is essential, as diastolic dysfunction was found to be a strong predictor of death in SSc [5]. Many SSc patients have smoldering, subclinical cardiac fibrosis, and even when symptomatic, the symptoms may be nonspecific (e.g., poor exercise tolerance and/or lack of functional capacity). Endomyocardial biopsy with histopathological analysis of myocardial tissue is the current gold standard for diagnosing myocardial fibrosis. While relatively safe, it is invasive and may be prone to sampling error, particularly in the setting of patchy, heterogeneously distributed myocardial fibrosis that occurs in SSc. Furthermore, echocardiography is limited in detecting diastolic dysfunction [49], and widely established biomarkers including BNP as well as its prohormone, N-terminal-proBNP, may be also elevated in SSc-PH [50–52] and are frequently normal in HFpEF (heart failure with preserved ejection fraction, secondary to diastolic dysfunction) [53]. Serum autoantibodies and clinical features are only marginally helpful in assessing the risk for cardiac fibrosis in SSc, with conflicting reports from multiple studies [54, 55]. Lastly, there is a lack of a consensus definition for what constitutes as SSc cardiac involvement [56]. Thus, there is a pressing need for validated, sensitive biomarkers to aid in detecting cardiac involvement in patients with SSc.

Periostin expression in cardiac tissue is strongly upregulated in various animal models of cardiac fibrosis [27, 57, 58] and after myocardial infarction and in heart failure [28]. In human failing hearts, cardiac periostin positively correlated with the degree of myocardial fibrosis as well as with left ventricular diastolic dimension [59]. Our study showed increased periostin deposition in a patchy distribution in the SSc cardiac tissue, which colocalized with collagen type I expression, but was also present in areas without any detectable collagen. This suggests that the accumulation of periostin in cardiac tissue of SSc patients may be an early event, supporting the notion that periostin may be a marker of myofibroblasts activation in the SSc heart, similar to findings from lineage tracing in mice [26].

Although periostin is considered a reliable marker of cardiac fibrosis, few studies have focused on the change in circulating periostin in cardiac fibrosis. Higher serum periostin level was related to increased composite cardiovascular events [60], and circulating periostin was higher in patients with severe dilated cardiomyopathy and correlated to the degree of diastolic dysfunction [61]. Our current study shows a direct correlation between circulating periostin levels in SSc patients and several echocardiography parameters related to the mass and size of the left ventricle, including LV mass, LV mass index, and LVEDD that can reflect myocardial fibrosis and ventricular remodeling. While these parameters can still be within normal range for many patients with subclinical cardiac involvement, the direct correlation suggests that changes in the size and mass of the heart can be reflected in circulating periostin levels, making it a promising biomarker for disease progression in SSc cardiomyopathy. Further studies will be required to assess its utility in identifying cardiac involvement in SSc.

Our study has limitations. First, our study had 22 healthy controls compared to 106 SSc patients. However, other studies have also shown significantly increased levels of both dermal and circulating periostin levels in SSc patients compared to healthy controls [13, 19, 20]. Secondly, our findings are based on retrospective observational data that is dictated by usual clinical practice which may lead to inconsistencies in data availability in addition to issues with timing. Furthermore, because our database spanned nearly two decades, there certainly may have been changes to clinical practice which may have evolved over time and may have impacted our findings. Thirdly, while our findings suggest strong associations between periostin and cardiac fibrosis on immunostaining as well as strong correlations with structural measurements on echocardiography, there is a lack of information regarding subclinical cardiac involvement that would be otherwise undetectable by routine echocardiography. Another caveat is the lack of available clinical information regarding the presence of scleroderma cardiac involvement in patients enrolled in our database. When cardiac disease is advanced enough to cause symptoms, patients complain of shortness of breath, which can also be seen with ILD and PAH. It is thus not surprising that the degree of shortness of breath as assessed by NYHA functional classification did not correlate with the levels of periostin in our study. Cardiac involvement in the form of fibrosis with diastolic dysfunction is frequently missed by conventional echocardiography and measurement of serum BNP or proNTBNP levels, all of which can still be within normal limits. No consensus currently exists in the literature regarding the definition of SSc involvement in the heart, and the reported range of cardiac involvement in SSc patients is very wide, depending on the definition used in a particular study [56]. Autopsy studies show almost uniform involvement in up to 80% of patients, mostly in the form of cardiac fibrosis [62, 63], while other studies have shown lower incidence [64]. Nevertheless, it is uniformly accepted that the rate of cardiac fibrosis and diastolic dysfunction in SSc are underestimated [5]. This is also supported by our findings in the cardiac tissue from deceased SSc patients. It is unknown whether these patients had cardiac involvement, but they showed evidence of increased periostin expression, compared to control cardiac tissue, which exhibited only scarce, or no periostin deposition. Future work will need to prospectively investigate periostin levels in SSc patients with assessments of both clinical and subclinical measurements of cardiac function in a time-controlled manner.

## Conclusion

In summary, we showed that circulating periostin levels directly correlated to the degree of skin fibrosis and were associated with disease duration in patients with SSc. Furthermore, we found that periostin was highly expressed in SSc cardiac tissue and its serum levels correlate to LV mass and LV mass index. To our knowledge, this is the first study to show that periostin is elevated in SSc cardiac tissue, suggesting that it may be a marker to predict ventricular remodeling due to increased ECM deposition by activated cardiac fibroblasts in SSc.

## Data Availability

All data generated or analyzed during this study are included in this published article. The datasets generated and/or analyzed during the current study are the subject of ongoing analysis and will be available from the corresponding author on reasonable request and subject to ethical approval. Some restrictions may apply pursuit to ongoing analysis.

## Abbreviations

ACR: American College of Rheumatology
ANOVA: Analysis of variance
ASMA: Alpha smooth muscle actin
BNP: Brain natriuretic peptide
BSA: Bovine serum albumin
CAT scan: Computed tomography scan
DAPI: Diamidino-2-phenylindole
dcSSc: Diffuse cutaneous systemic sclerosis
D_LCO_: Diffusing capacity for carbon monoxide
ECM: Extracellular matrix
ELISA: Enzyme-linked immunosorbent assay
EULAR: European League Against Rheumatism
FAK: Focal adhesion kinase
FEV1: Forced expiratory volume
FVC: Forced vital capacity
FV10i: Fluoview
IL: Interleukin
ILD: Interstitial lung disease
IPF: Idiopathic pulmonary fibrosis
IVS: Interventricular septum
lcSSc: Limited cutaneous systemic sclerosis
LV: Left ventricular
LVDD: Left ventricular diastolic diameters
LVEDD: Left ventricular end-diastolic diameters
LVM: Left ventricular mass
LVMI: Left ventricular mass index
LVSD: Left ventricular systolic diameters
mPAP: Mean pulmonary arterial pressure
mRSS: Modified Rodnan skin score
NDRI: National Development and Research Institutes
NT-proBNP: N-terminal -pro hormone brain natriuretic peptide
ON: Overnight
PAH: Pulmonary arterial hypertension
PAWP: Pulmonary arterial wedge pressure
PBS: Phosphate buffered saline
PECAM-1: Platelet and endothelial cell adhesion molecule 1
PFT: Pulmonary function test
PI3K: Phosphoinositide 3-kinase
*p* value: Probability value
PVR: Pulmonary vascular resistance
PW: Posterior wall
SCaR: Scleroderma clinic at rheumatology
SSc: Scleroderma, systemic sclerosis
CMP: Cardiomyopathy
Th-2: T-helper type-2
TGFβ: Transforming growth factor beta
