# Correction: Periostin overexpression in scleroderma cardiac tissue and its utility as a marker for disease complications

**DOI:** 10.1186/s13075-023-02993-0

**Published:** 2023-01-23

**Authors:** Fatima El-Adili, Justin K. Lui, Mortada Najem, Giuseppina Farina, Maria Trojanowska, Flora Sam, Andreea M. Bujor

**Affiliations:** 1 Division of Rheumatology, Boston University School of Medicine, Boston, MA USA; 2 Whitaker Cardiovascular Institute, Boston University School of Medicine, Boston, MA USA; 3 Arthritis and Autoimmune Diseases Center, Boston University, 72 E Concord St, Evans 501, Boston, MA 02118 USA; 4 The Pulmonary Center, Boston University School of Medicine, Boston, MA USA

**Correction: Arthritis Res Ther 24, 251 (2022)**

**https://doi.org/10.1186/s13075-022-02943-2**

Following publication of the original article [1], an error was identified in the Funding section.

The updated funding is given below and the changes have been highlighted in bold.

## Funding

A.M.B. was supported by Tobe and Stephen E. Malawista, MD, Endowment in Academic Rheumatology, Rheumatology Research Foundation and by NIH/NHLBI HL155955-01A1. F.E. is supported by NIH 5T32HL007224-45, M.T. is funded by NIAMS P50 AR060780, F.S. **is funded by NIH/NHLBI R01HL145985**, and J.K.L is funded by NIH/NHLBI1F32HL156614-01.

The original article [1] has been updated.
